# *Moraxella lacunata* infection accompanied by acute glomerulonephritis

**DOI:** 10.1515/med-2020-0234

**Published:** 2020-10-01

**Authors:** Nami Sawada, Tamaki Morohashi, Tomokazu Mutoh, Tsukasa Kuwana, Junko Yamaguchi, Kosaku Kinoshita, Ichiro Morioka, Hiroyuki Hao

**Affiliations:** Department of Acute Medicine, Division of Emergency and Critical Care Medicine, Nihon University School of Medicine, 30-1 Oyaguchi Kamimachi, Itabashi-ku, Tokyo, 173-8610, Japan; Department of Pediatrics and Child Health, Nihon University School of Medicine, 30-1 Oyaguchi Kamimachi, Itabashi-ku, Tokyo, 173-8610, Japan; Department of Pathology, Division of Human Pathology, Nihon University School of Medicine, 30-1 Oyaguchi Kamimachi, Itabashi-ku, Tokyo, 173-8610, Japan

**Keywords:** *Moraxella lacunata*, children, complement activity, hemodialysis, sepsis

## Abstract

*Moraxella lacunata* (*M. lacunata*) is a Gram-negative bacterium, which rarely causes serious infection. This is a rare case report of acute glomerulonephritis diagnosed by pathological findings in a child accompanied by *M. lacunata* infection. The patient showed hematuria, proteinuria and hyperkalemia requiring emergency hemodialysis. After hospitalization, *M. lacunata* bacteremia became apparent. Pathological findings showed an increase in glomerulus inflammatory cells and glomerular C3 deposition was observed in the renal tissue biopsy. Final diagnosis was endocapillary proliferative glomerulonephritis. Clinical reports of *M. lacunata* infection requiring emergency hemodialysis in children are rare. Previous reports have suggested that lowered immune competency with chronic kidney disease may be a risk factor associated with serious invasive cases of *M. lacunata* infection. However, detailed clinical laboratory data and pathological findings have not been identified in previous case reports. Our case directly indicated complement activity and acute glomerulonephritis with *M. lacunata* infection. Although there are various causes for acute glomerulonephritis, infection-related glomerulonephritis (IRGN) is an important concept. *M. lacunata* infection might have a potential risk for IRGN with dysregulation of complement activity leading to serious and invasive clinical conditions than previously considered.

## 1 Introduction

*Moraxella lacunata* (*M*. *lacunata*) is a Gram-negative bacterium found in healthy commensal flora of the upper respiratory tract. *M*. *lacunata* rarely causes infection such as conjunctivitis, keratitis endophthalmitis, endocarditis and septic arthritis [1,2]. *M. lacunata* usually responds to antibiotics such as beta-lactams, aminoglycosides and quinolones [2]. According to a review of previous case reports, cases of systemic *M. lacunata* infection with acute kidney injury requiring emergency hemodialysis are few [2]. Moreover, in the previous reports, detailed clinical information and pathology of kidney injury have not been clarified, and there are few reports on acute glomerulonephritis in child patients with *M. lacunata* infection. Consequently, this is considered to be a rare case report demonstrating clinical laboratory data and pathological findings of a patient with *M. lacunata* infection (infection-related glomerulonephritis [IRGN] [3,4]) in children. In addition, this case report includes a literature review of systemic *M. lacunata* infections with acute kidney injury.

## 2 Case report

This is a case of 12-year-old boy with no noteworthy medical history. Ten days before hospital admission, the patient experienced abdominal pain and diarrhea. A day before admission, enlarged lymph nodes that showed tenderness were observed on the right side of the neck. Palpebral edema, hematuria and hyperkalemia (serum potassium: 7.0 mmol/L) were diagnosed at the initial physical examination by the primary care doctor. The patient received a calcium gluconate (0.085 g) IV and three glucose–insulin infusion (40 mL of 50% glucose and 8 U of regular insulin) IVs. After these IV treatments, serum potassium level decreased to 5.8 mmol/L, temporarily. Symptoms of the respiratory tract such as pharyngitis were not observed and rapid antigen detection test for group A *Streptococcus* by throat swab was negative.

The patient visited the hospital emergency department due to kidney injury with recurrence of hyperkalemia. Serum sodium and potassium were 138 and 7.7 mmol/L, respectively. Tented T waves on his ECG and acute lymphadenitis in the neck were also observed. Blood pressure at hospital arrival was 165/93 mm Hg, pulse was 78 beats per minute and body temperature was 37.6°C on admission. On physical examination, bilateral eyelid edema and lymph node enlargement with tenderness in the right front area of the neck were observed. However, sore throat, skin lesions, abdominal tenderness, hepatosplenomegaly and bilateral inguinal lymphadenopathy were not observed. In addition, insect bites and signs of systemic infection were also not observed.

Laboratory data upon admission, as shown in Table 1, included white blood cell count: 12,700/μL; hemoglobin: 8.1 g/dL; and platelets: 3,16,000/μL. Blood urea nitrogen was 77.9 mg/dL and serum creatinine concentration was 1.15 mg/dL (the normal range of his age is 0.40–0.61 mg/dL). The patient was placed in the admitted intensive care unit (ICU) due to refractory hyperkalemia with acute kidney injury (estimated glomerular filtration rate was calculated to be 59 mL/min/1.73 m^2^ and urine output was <5 mL/h for the first 24 h with a median of 15 mL/h between 6 and 48 h after admission). Both Acute Kidney Injury Network and Kidney Disease: Improving Global Outcomes criteria indicated stage 2. A second rapid antigen detection test for group A *Streptococcus* by the throat swab was negative. Antistreptolysin-O (ASO) titre was 689 IU/mL (normal range < 300) and antistreptokinase (ASK) titre was 5,120 IU/mL (normal range <5,120).

**Table 1:** Laboratory data on admission

WBC (4.0–8.0 × 10^3^/μL)	12.7	CRP (<0.2 mg/dL)	0.78
Neurocyte (%)	78.1	Presepsin (<500 pg/mL)	159
Lymphocyte (%)	15.9	Procalcitonin (<0.5 ng/mL)	0.24
Monocyte (%)	3.9	FDP (<5 μg/mL)	9.3
Eosinocyte (%)	1.9	D-dimmer (<1 μg/mL)	4.90
RBC (4.5–5.5 × 10^6^/μL)	3.14	Na (136–148 mmol/L)	138
Hgb (14–17 g/dL)	8.1	K (3.6–5.0 mmol/L)	7.7
Hct (40–50%)	25.0	Cl (98–109 mmol/L)	109
Platelet (158–348 × 10^3^/μL)	316	Ca (8.8–10.8 mg/dL)	8.8
T-Bil (0.3–1.2 mg/dL)	0.53	P (2.4–4.5 mg/dL)	5.5
D-Bil (0.05–0.4 mg/dL)	0.14	Mg (1.7–2.3 mg/dL)	2.8
AST (8–38 U/L)	20		
ALT (4–44 U/L)	10	Urine analysis	
LD (106–220 U/L)	241	Urine pH	5.5
ALP (117–335 U/L)	390	Urine gravity	1.010
G-GT (12–73 U/L)	11	Urine protein	3+
CK (54–253 U/L)	52	Urine occult blood	3+
TP (6.5–8.0 g/dL)	7.4	Urine sugar	—
Albumin (3.8–5.3 g/dL)	2.8		
Blood sugar (70–110 mg/dL)	87		
UN (8–19 mg/dL)	77.9		
Creatinine (0.40–0.61 mg/dL)	1.15		

Other clinical data including immunological test data are listed in Table 2. Serum concentrations of complement (C) 3, C4 and 50% hemolytic complement activity (CH50) were 4 mg/dL (normal range: 73–138), 12 mg/dL (normal range: 11–31) and <10.0 U/mL (normal range: 30–45), respectively. Immunoglobulin values of IgG (normal range: 861–1,747 mg/dL), IgA (normal range: 93–393 mg/dL) and IgM (normal range: 33–83 mg/dL) were 2,649, 330 and 99 mg/dL, respectively. Other immunological tests for Sjögren’s syndrome, lupus erythematosus and antineutrophil cytoplasmic antibody (ANCA)-associated vasculitis were negative. Urine sediment contained white blood cells, and urine blood and urine protein were both 3+. A third rapid antigen detection test for group A *Streptococcus* by the throat swab on the second hospital day was also negative. Only normal commensal flora grew in the throat culture, and group A beta-hemolytic *Streptococcus* did not appear.

**Table 2:** Immunological test

IgG (861–1,747 mg/dL)	2,649	CMV IgG	Negative
IgA ( 93–393 IU/L)	330	CMV IgM	Negative
IgM (33–183 mg/dL)	99	EBV-VCA IgM	Negative
C3 (73–138 mg/dL)	4	EBV-VCA IgG	Negative
C4 (11–31 mg/dL)	12	Anti-GBM antibody	Negative
CH50 (30–45 U/mL)	<10.0	Anti-nuclear antibody	Negative
		Anti-SS-A/B antibody	Negative
ASO (<300 IU/mL)		Anti-dsDNA antibody	Negative
On adm	689	Anti-PR3-ANCA antibody	Negative
2 wks after adm	922	MPO-ANCA antibody	Negative
4 wks after adm	369	Anti-CCP antibody	Negative
12 wks after adm	236	Human parvovirus b19 antigen	Negative
ASK (<5120 IU/mL)		HBs antibody	Negative
On adm	5,120	HCV antibody	Negative
2 wks after adm	5,120	*Treponema pallidum*	Negative
4 wks after adm	NA	HIV	Negative
12 wks after adm	640	Filaria	Negative
		Malaria	Negative
		T-SPOT test	Negative

adm: admission; wks: weeks; NA: not available; C: complement; ASO: antistreptolysin‐O; ASK: antistreptokinase; HBs: hepatitis B surface; anti-SSA/B autoantibodies: anti-Sjögren’s-syndrome-related antigen A/B; GBM: glomerular basement membrane; Antineutrophil cytoplasmic antibodies (ANCA, MPO, PR3); anti-CCP: anti-cyclic citrullinated peptide; anti-dsDNA: anti-double stranded DNA; ANCA: antineutrophil cytoplasmic antibody; MPO: myeloperoxidase; PR3-ANCA: proteinase 3-antineutrophil cytoplasmic antibody; T-SPOT: a type of enzyme-linked immune absorbent spot (ELISpot) assay used for tuberculosis diagnosis, and belongs to the group of INF gamma release assays.

Antibiotic therapy (Ampicillin/Sulbactam: ABPC/SBT 3g q6hr) for acute lymphadenitis accompanied with leukocytosis was initiated. Emergency hemodialysis was performed for hyperkalemia showing tented T waves on the ECG. Hyperkalemia gradually improved after hemodialysis. Three days after admission, urine output exceeded 0.5 mL/kg/h and blood potassium level decreased to 4.7 mmoL/L (Table 3), so continuous hemodialysis was discontinued. However, body temperature increased to 40.0°C (serum white blood cell count was 28,200/μL, C-reactive protein [CRP] was 6.92 mg/dL, quick SOFA was 2; respiration rate was 24/min, heart rate was 132/min, and his consciousness level was lethargic). On day 4, *M. lacunata* was detected by two sets of blood cultures, which were collected on the third day during the fever. Consequently, *M. lacunata* bacteremia became apparent. *M. lacunata* infection was speculated to originate with lymphadenitis, as conjunctivitis, keratitis endophthalmitis, endocarditis and septic arthritis were ruled out according to other laboratory data and transesophageal echocardiography during clinical course. In addition, antibiotics were switched to Cefotaxime: CTX 1g q12hr on day 8.

**Table 3:** Trends of laboratory data after admission

Admission days	1	2	3	4	6	8	10	15	25
WBC (4.0–8.0 × 10^3^/μL)	12.7	11.2	11.5	28.2	14.2	11.4	9.1	7.6	7.2
CRP (<0.2 mg/dL)	0.78	0.74	0.84	6.92	3.71	2.19	1.06	1.02	0.57
Platelet (158–348 × 10^3^/μL)	316	289	341	297	219	225	215	387	260
T-Bil (0.3–1.2 mg/dL)	0.53	0.45	0.51	0.49	0.40	0.32	0.34	0.24	0.22
Creatinine (0.40–0.61 mg/dL)	1.15	1.01	1.59	2.02	1.22	0.79	0.69	0.64	0.73
K (3.6–5.0 mmol/L)	7.7	5.7	4.7	4.4	4.5	4.5	4.6	4.3	4.4

Admission days	1	7	30	90	150	270
C3 (73–138 mg/dL)	4.0	7.0	44.0	72.0	74.0	79.0
CH50 (30–45 U/mL)	10	10.4	43.5	51.4	56.7	58.2

C: complement.

**Figure 1:**
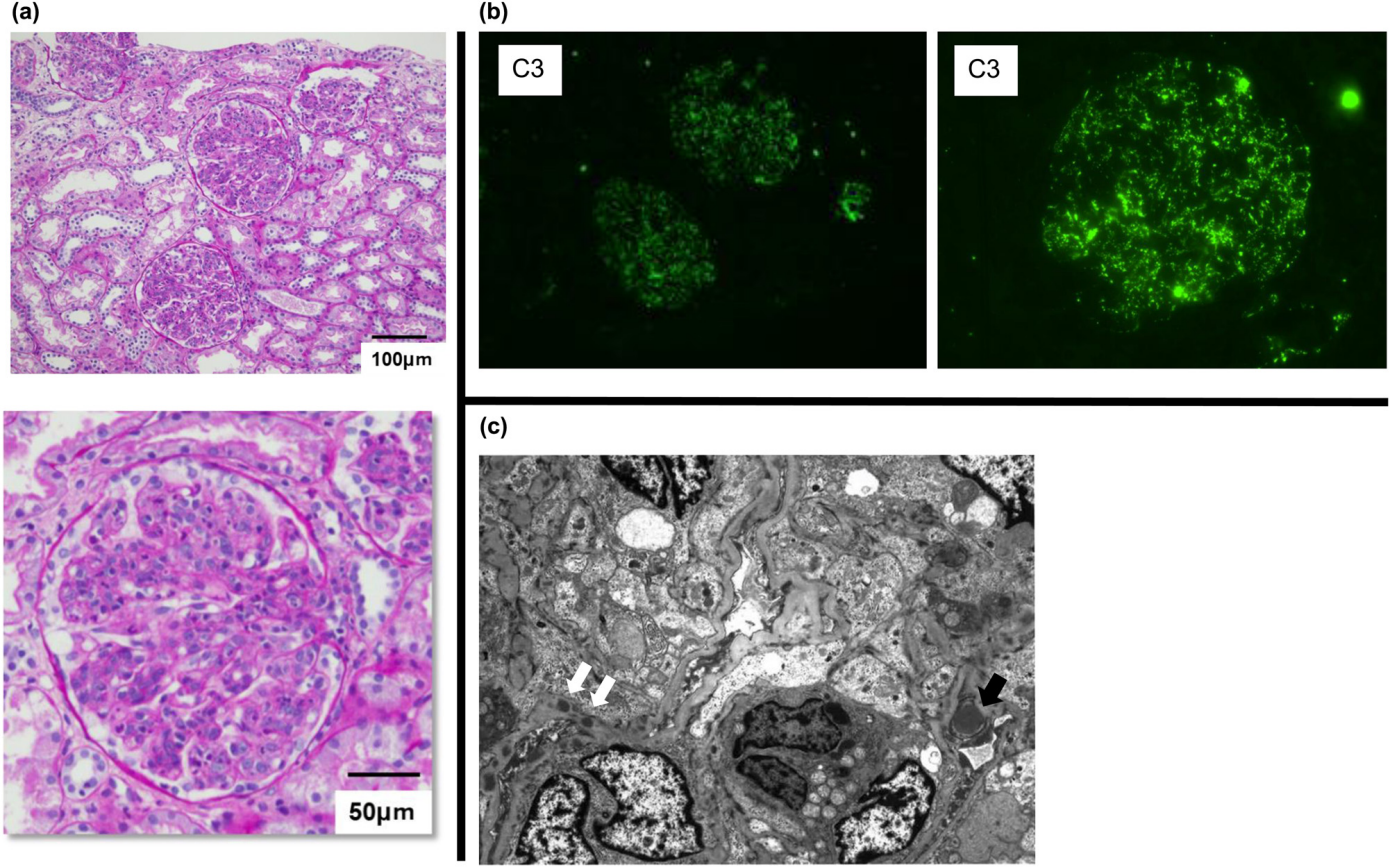
Pathological observation by percutaneous renal biopsy. (a) PAS stain. (b) Fluorescent antibody method. (c) Electronic microscope. According to pathological observation, glomerulus inflammatory cells infiltrating into the glomerular tuft increased. Glomerular C3 deposition (starry sky pattern) was observed by fluorescent antibody method. Electronic microscope finding of a portion of a glomerulus. Subepithelial dense deposit (hump; black arrow) and intramembranous deposits (white arrows) are shown. C3: complement 3.

Percutaneous renal biopsy was performed on day 11 (Figure 1). Pathological findings indicated increased infiltration of inflammatory cells into the glomerular tuft and narrowing of the vascular lumen. Immunofluorescence studies were also performed for IgG, IgA, IgM, C3, C4 and C1q (Complement component 1q) antibodies. Glomerular C3 deposition (starry sky pattern) was observed, but IgG and other immunoglobulin depositions were not detected in the renal tissue biopsy by the fluorescent antibody method. Final pathological diagnosis was endocapillary proliferative glomerulonephritis and was clinically consistent with acute kidney injury.

The patient did not show any serologic evidence preceding *streptococcal* infection, however, sequential changes in serum ASO or ASK titre indicated a peak infection around 2 weeks after hospital admission and then gradually decreased during next 12 weeks (Table 2).

The patient was moved from ICU to the general ward and antibiotics were discontinued on day 15. Urine protein and hematuria were still positive on day 20, so oral administration of prednisolone (60 mg/day) was initiated and reduced to 30 mg/day on day 34, to 15 mg/day on day 48, to 5 mg/day on day 62 and was discontinued on day 76. C3 was gradually normalized on day 30 (Table 3). He was finally discharged on day 42 with good recovery, negative urine protein on day 96 and recovery of hematuria on day 162.


**Informed consent:** Written informed consent was obtained from the patient’s family before hospital discharge for this case report.

## 3 Discussion

Previously, some clinical case reports on patients with *M. lacunata* infection have been published. Among these, three cases of chronic kidney disease (end-stage kidney disease [2], Lupus nephritis [5] and diabetic nephropathy [6]) with hemodialysis and one case of *M. lacunata* infectious arthritis with a nephrotic syndrome [7] were included. However, a clinical case where a patient who visited the hospital emergency due to acute kidney injury requiring emergency hemodialysis has not been reported. Our patient had no noteworthy medical history, such as chronic kidney disease, and was admitted with acute kidney injury. Moreover, *M. lacunata* infection became apparent after hospitalization. Previous case reports have considered that chronic kidney disease may be a risk factor associated with serious invasive cases of *M. lacunata* infection, suggesting immune suppression or activation of a complement pathway [5,8,9]. However, activation of a complement pathway has not been clinically proven and detailed laboratory data and pathological findings have not been provided in previous case reports. Our patient directly demonstrated activation of the complement pathway and was diagnosed with endocapillary proliferative glomerulonephritis. Sepsis-related acute kidney injury is commonly considered to affect renal vasoconstriction and progress to acute tubular necrosis [10]. According to pathological findings in this case, however, infiltration of inflammatory cells into the glomerular tuft with deposition of C3 was indicated and final pathological diagnosis was acute endocapillary proliferative glomerulonephritis accompanying *M. lacunata* infection. In this case, clinically low serum C3 could also be identified, which supports the hypothesis of previous case reports [5,8,9].

Acute glomerulonephritis develops from various causes and infections are an important cause in the concept of IRGN [3,4]. Hypocomplementemia is exhibited with IRGN, and C3 is depressed with or without depression of C4 [11]. In this case, symptoms of bacterial infection were present with depression of C3 when acute glomerulonephritis was diagnosed. As no clinical evidence of group A *Streptococcus* infection were observed in his past history, this case is considered to be clinically consistent with IRGN. Common cause of acute glomerulonephritis in children is now recognized as an immunologically mediated complication [12]. Initial clinical symptoms are a sore throat such as pharyngitis, edema and hematuria, and hypertension. These are typical symptoms for acute glomerulonephritis diagnosis. This case deteriorated into acute kidney injury requiring emergency hemodialysis in a 2-week clinical course. *M. lacunata* infection may carry the risk of dysregulation of complement activity, leading to serious and invasive clinical conditions which interact with each other to a greater extent than previously considered [1,2,7]. Although various bacteria may be the cause of IRGN and the fact that some genetic factors may affect IRGN have been considered [4], whether *M. lacunata* itself is a potential cause for IRGN remains unclear. However, it is of critical concern if *M. lacunata* infection could be a risk of IRGN, affecting the activation of the complement pathway.

Some reports have indicated that invasive case of *M. lacunata* infection is observed in patients with chronic disease, such as heart disease, arterial hypertension and chronic renal disease [1,2,5,7]. In general, Ampicillin/Sulbactam: ABPC/SBT or beta-lactam antibiotics Cephalosporin, such as Cefotaxime and Ceftriaxone, are effective for *M. lacunata* bacteria infection itself, except for beta-lactamase-producing *M. lacunata* [2,13]. The combination of aminoglycoside with β-lactam antibiotics is recommended for infective endocarditis of specific bacteria (*Enterococcus* and *Staphylococcus* in artificial valve patients) [14], and this combination antibiotics therapy may be useful in patients with infective endocarditis of *M. lacunata* as well [2,15]. Although dependent on any medical history of chronic disease or systemic complications, outcome of infection/bacteremia caused by *M. lacunata* itself is relatively favorable [2,15]. In our case, infectious symptoms promptly improved after antibiotic therapy, but additional treatment for renal dysfunction was needed. Rapid clinical evaluation to determine the presence of organ dysfunction is required in cases of serious invasive disease of *M. lacunata* bacteria infection in addition to antibiotic therapy.

In conclusion, this is a rare case report of *M. lacunata* infection with acute kidney injury requiring emergency hemodialysis, which was diagnosed as endocapillary proliferative glomerulonephritis. This also directly indicated clinically lowered immune competency in serious, invasive cases of *M. lacunata* infection.
